# ILSI Southeast Asia symposium: prevalence, risk factors, and actions to address gestational diabetes in selected Southeast Asian countries

**DOI:** 10.1038/s41430-020-00838-6

**Published:** 2021-02-02

**Authors:** Maria Sofia Amarra, Mary Foong-Fong Chong, Vitaya Titapant, Charintip Somprasit, Jossie Rogacion, Rima Irwinda, Trang Nguyen Khanh Huynh, Sivalingam Nalliah

**Affiliations:** 1 Department of Food Science and Nutrition, College of Home Economics, University of the Philippines Diliman, Quezon, Philippines; 2 Saw Swee Hock School of Public Health, National University of Singapore, Singapore, Singapore; 3 Department of Obstetrics and Gynecology, Faculty of Medicine, Siriraj Hospital Mahidol University, Bangkok, Thailand; 4 Department of Obstetrics and Gynecology, Faculty of Medicine, Thammasat University, Bangkok, Thailand; 5 Department of Pediatrics, University of the Philippines, Manila, Philippines; 6 Department of Obstetrics and Gynecology, Faculty of Medicine, Universitas Indonesia, Jakarta, Indonesia; 7 Department of Obstetrics and Gynecology, Pham Ngoc Thach University of Medicine, Ho Chi Minh, Vietnam; 8 Division of Obstetrics & Gynaecology, Clinical School, International Medical University, Seremban, Malaysia

**Keywords:** Risk factors, Nutrition

## Introduction

Gestational diabetes mellitus (GDM) is increasing, particularly in Southeast Asia. This paper presents findings from a symposium organized by the International Life Sciences Institute Southeast Asia (ILSI SEA) which discussed the growing issue of gestational diabetes and how it can be addressed in the region. GDM together with diabetes in pregnancy (DIP) both fall under the definition of hyperglycemia in pregnancy [1]. GDM is defined as diabetes diagnosed for the first time during pregnancy mostly after 24 weeks gestation. DIP is defined as pregnant women having higher oral glucose tolerance test (OGTT) results [1]. Table 1 shows the WHO criteria that distinguish GDM and DIP [2].

**Table 1:** WHO criteria for GDM and DIP [2].

	Gestatational diabetes mellitus (GDM)	Diabetes in pregnancy (DIP)
Requirement for classification	One or more of the following criteria are met at any time in pregnancy	One or more of the following criteria are met (for both pregnant and non-pregnant women)
Fasting plasma glucose	5.1–6.9 mmol/L (92–125 mg/dL)	≥7.0 mmol/L (126 mg/dL)
1-h plasma glucose	≥10.0 mmol/L (180 mg/dL) following a 75 g oral glucose load	–
2-h plasma glucose	8.5–11.0 mmol/L (153–199 mg/dL) following a 75 g oral glucose load	≥11.1 mmol/L (200 mg/dL) following a 75 g oral glucose load
Random plasma glucose	n/a	≥11.1 mmol/L (200 mg/dL)

– no established criteria for the diagnosis of diabetes based on the 1-h post-load value, *n/a* not applicable.

Women with hyperglycemia in pregnancy have a higher risk of developing diabetes over 15 years after the index pregnancy. It was estimated that 75–90% of cases of hyperglycemia in pregnancy are GDM [1]. Although data are lacking, GDM prevalence is thought to parallel the rising incidence of type 2 diabetes mellitus in the background population [1]. Table 2 shows the 2019 prevalence of diabetes and undiagnosed diabetes in Southeast Asian countries [1].

**Table 2:** Age-adjusted comparative prevalence of diabetes in adults aged 20,979 years (2019) by country [1].

	Diabetes age-adjusted comparative prevalence (%) in adults 20–79 year (95% CI)	Number of adults 20–79 year with undiagnosed diabetes in 1000 s (95% CI)
Malaysia	16.7 (14.9–19.2)	1841.3 (1652.2–2125.9)
Brunei	13.3 (9.3–17.6)	18.7 (14.0–24.3)
Philippines	7.1 (5.6–8.9)	2662.3 (2122.7–3360.4)
Thailand	7.0 (5.4–8.1)	1868.0 (1444.1–2145.2)
Cambodia	6.3 (4.9–11.0)	268.1 (199.5–530.4)
Indonesia	6.3 (5.4–6.8)	7870.1 (6789.6–8509.8)
Lao PDR	6.3 (4.9–11.0)	102.3 (78.2–192.8)
Vietnam	6.0 (4.9–8.1)	2017.7 (1646.4–2670.9)
Singapore	5.5 (4.7–6.3)	346.0 (300.8–389.0)
Myanmar	3.9 (3.0–5.9)	684.7 (537.6–1001.6)

The objectives of this ILSI SEA symposium were to: (i) assess the prevalence of GDM in selected countries (Malaysia, Singapore, Indonesia, Thailand, Philippines, Vietnam) and identify nutritional and other risk factors; (ii) identify country actions to address GDM and challenges in implementing these actions; (iii) recommend measures to prevent/reduce the prevalence of GDM in Southeast Asia.

## Results

### Prevalence of GDM in selected Southeast Asian countries

**Fig. 1:**
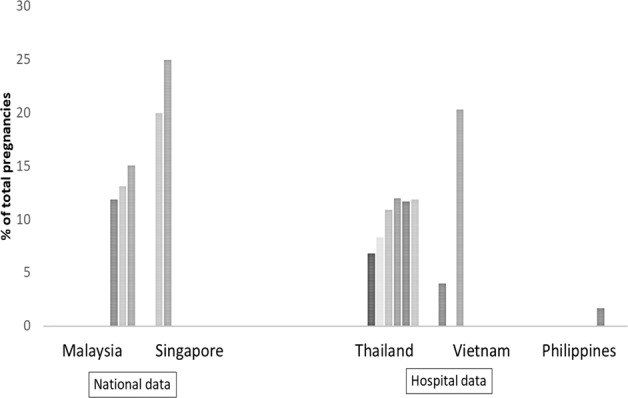
Prevalence of GDM in Southeast Asian countries based on existing data.

Figure 1 shows the prevalence of GDM in selected Southeast Asian countries based on existing data.

Prevalence estimates ranged from 1.7% to 25%, based on varying criteria. Only Singapore and Malaysia have national estimates for GDM prevalence. Vietnam, Indonesia, Thailand, and the Philippines only have hospital-based data. Based on national data, Singapore has a higher prevalence of GDM than Malaysia (Fig. 1). Based on hospital data, Vietnam has the highest prevalence, followed by Thailand, and lastly the Philippines. Using hospital data, Purnamasari et al. [3] reported that the prevalence of GDM in Indonesia ranged from 1.9% to 3.6% but the period was not specified. All countries showed an increasing trend in GDM prevalence over time.

Limitations of the data are the fact that each country used different criteria to diagnose GDM which limits comparability. Most had small sample sizes derived from hospital data. There is no uniformity in employing criteria for the diagnosis of GDM among the nations. The International Association of Diabetes and Pregnance Study Groups (IADPSG), American Diabetes Association (ADA), and WHO criteria were employed. Malaysia, Singapore, and Vietnam reported higher prevalence when IADPSG criteria were used, compared to other criteria.

### Nutritional risk factors for GDM in selected Southeast Asian countries

Table 3 summarizes the nutritional and non-nutritional risk factors for GDM based on existing evidence.

i. *Anthropometric measurement*. In all countries, pre-conception overweight and obesity were important risk factors for GDM [4–6]. The lowest BMI level associated with GDM occurred among Vietnamese women (BMI ≥ 21), while the highest level occurred among Malaysian women (BMI > 27).
ii. *Dietary intake*. In Singapore and Indonesia, dietary patterns, as well as specific foods and nutrients, were shown to be risk factors for GDM. In Singapore, the Growing Up in Singapore Towards Healthy Outcomes (GUSTO) cohort study identified two key dietary patterns [7]. The first dietary pattern was a vegetable, fruits, rice-based pattern characterized by high consumption of vegetables, fruits, whole grain bread, nuts and seeds, white rice, and lower consumption of carbonated and sweetened drinks, burgers, meat, fried potatoes. The second dietary pattern was seafood, noodle, and soup-based pattern characterized by high consumption of soup, fish and seafood products, noodles, red meat, seafood, soya-based gravy, and lower consumption of legumes/pulses, ethnic bread, rice, curry gravy, other grains. Mothers following the seafood and noodle dietary pattern had a lower risk of GDM compared to those consuming the rice-based pattern.
  In Indonesia, a retrospective study [8] showed that the risk of prediabetes and GDM increased among respondents with a history of less fiber consumption (OR 2.35, 95% CI 1.12, 4.94) and less coffee consumption (OR 2.41, 95% CI 1.10, 5.25).
iii. *Micronutrient status*. The Singapore GUSTO study showed that the combination of high folate and vitamin B12 (cobalamin) deficiency was associated with higher GDM risk, compared to those with insufficient B12 and low concentrations of folate. If women were sufficient in B12, no significant associations with the risk of GDM were observed with increasing folate concentrations. The results suggest that an imbalance in the two B-vitamins may be responsible for glucose intolerance. Adjustment for pre-pregnancy BMI attenuated the effect of different concentrations of B12 on the prevalence of GDM, indicating that BMI explains part of the association of vitamin B12 with plasma glucose concentrations and GDM.
  The exact mechanism of this phenomenon is unclear. One theory is that when B12 is insufficient, the conversion of 5-methyltetrahydrofolate to tetrahydrofolate is inhibited. This impairs the production of purines and pyrimidines for DNA/RNA synthesis, resulting in impaired mitochondrial DNA which in turn is correlated with the development of insulin resistance. Insufficient B12 was also shown to increase adiposity during pregnancy, which may also impair insulin signaling [9]. These findings suggest that other micronutrient deficiencies should be considered when supplementing mothers during pregnancy and that the combination of high folate with low B12 can lead to metabolic consequences.
  In Indonesia, Wibowo et al. [10] examined 234 pregnant women in the first trimester of pregnancy. Prevalent micronutrient deficiencies were vitamin A (69.7% of women), vitamin D (99.6%), and zinc (81.2%). Vitamin D and zinc deficiencies were shown to increase the risk of GDM.
iv. *Combined diet and exercise regimen*. Trang et al. [11] demonstrated the beneficial effect of combined physical activity (walking 30 min/day) and medical nutrition therapy (MNT) (hospital daily menu only with no other food and drinks) on blood sugar levels in Vietnamese pregnant women with GDM. The proportion of women with stable blood glucose levels increased from 71.2% to 87.2% after 3 and 7 days, respectively (RR for blood glucose stability = 2.67, 95% CI 1.03, 6.92; *p* < 0.05).

**Table 3:** Nutritional and non-nutritional risk factors for GDM based on available studies.

Risk factor	Malaysia	Singapore	Indonesia	Thailand	Vietnam	Philippines
*Nutritional risk factors*						
Anthropometric measurement	BMI > 27 associated with higher risk	Overweight and obesity associated with higher risk	Overweight and obesity (BMI > 25) associated with higher risk	Overweight and obesity associated with higher risk [5]	BMI ≥ 21.1 associated with a higher risk [4]	Increasing BMI associated with higher risk [6]
Dietary intake	–	Seafood and noodle dietary pattern associated with lower risk compared to rice-based dietary pattern [7]	Factors associated with higher risk were less fiber intake (OR 2.35, 95% CI 1.12, 4.94) and less coffee (OR 2.41, 95% CI 1.10, 5.25) [8]	–	–	–
Micronutrient status	–	Combination of B12 deficiency and high folate associated with higher risk [13]	Vitamin D deficiency and zinc deficiency associated with higher risk; low serum iron associated with lower risk [10]	–	–	–
Diet and exercise regimen	–	–	–	–	Physical activity (30 min/day) combined with a strict diet was associated with improved blood glucose level [11]	–
Other factors (non-nutritional)	Previous history of GDM; First degree relative with diabetes mellitus; History of macrosomia (birth weight >4 kg); Bad obstetric history (unexplained intrauterine death, congenital anomalies, shoulder dystocia); Glycosuria ≥2 + on two occasions; Current obstetric problems (essential hypertension, pregnancy-induced hypertension, polyhydramnios, current use of corticosteroids)	–	–	Family history of diabetes mellitus (adjusted OR 1.86, 95% CI 1.38–2.51), Age > or = 30 years (adjusted OR 2.41, 95% CI 1.72–3.39); History of unexplained intrauterine fetal death (adjusted OR 4.30, 95% CI 2.04–9.04)	–	Family history of diabetes mellitus (OR 6.3); Use of hormonal contraception (OR 8.48).

– no information.

### Challenges in addressing GDM in Southeast Asia

At the regional level, the following common problems in the different countries were raised:

- *Lack of pre-conceptual screening*. Identifying and managing type 2 diabetes is end-stage management. Strategies to identify at-risk women should be put in place earlier.
- *Maternal weight gain and postpartum weight retention*. Obesity is common. Obese mothers have larger babies (almost double the size of normal weight) and a higher risk of preterm delivery due to complications associated with obesity.
- *Lack of longitudinal care*. The current “vertical point of care” approach is focused on reducing mortality and morbidity of the newborn and does not address long-term consequences in both mother and child.
- *Lack of emphasis on MNT*. MNT is the cornerstone in GDM management. The goal of MNT is to provide adequate calories and nutrients to meet the needs of pregnancy consistent with maintaining normoglycemia. However, there is no specific recommended dietary approach to manage GDM.
- *Absence of national GDM guidelines and inadequate implementation of existing national guidelines, if any*. Despite the presence of existing national guidelines for management in many countries, these are not closely adhered to across all health facilities within countries due to lack of manpower, budget, and low awareness of GDM among doctors and frontline health workers. In the absence of national guidelines, adherence to WHO guidelines were recommended.

## Discussion

The following measures were proposed to prevent/reduce GDM in the region.

### Adoption of universal screening and use of common screening methods and diagnostic criteria

A consensus is needed for common criteria to define GDM among Southeast Asian women. But whether this is possible to implement will require further studies as certain screening methods increased the prevalence of GDM, resulting in a heavy workload for the health care system. It was suggested that countries adopt the standard practice of having an OGTT during the second and third trimester and then once identified, refer to a dietitian for MNT and a physiotherapist for antenatal exercise. Universal screening rather than the selective screening of high-risk groups was recommended, to capture women without risk factors [12].

### Collection of nationwide epidemiological data by Southeast Asian countries

Except for Singapore and Malaysia, the rest of the countries do not have adequate data on the prevalence of GDM at the national level. Epidemiological data in the region can be established if a common definition for GDM is developed and Southeast Asian countries agree on how to screen and diagnose GDM. A national policy mandating doctors to report GDM cases to a national health registry or database will help. The registry can include BMI, family history, past medical history, etc. These data will allow countries to define the patterns, determinants, and distribution of disease. From there, plans can be made on how to address the problem of GDM. An ASEAN study to show the long-term health impact of GDM on the offspring is also needed.

### Implementation of the following actions at different stages

- During pre-conception, women should be encouraged to achieve an ideal BMI. Pregnancies should be planned, and pre-existing diabetes well-optimized prior to a planned pregnancy. Efforts are needed to increase awareness among mothers and families to ensure that the prevention of GDM is taken seriously. Currently, there is no clear guidance for mothers regarding the prevention of GDM.
- During pregnancy, women should be taught about proper dietary intake to prevent excessive gestational weight gain and weight gain monitoring. Timing and mode of delivery should be planned to reduce perinatal mortality and sudden intrauterine demise.
- After delivery, women should be encouraged to practice exclusive breastfeeding for the first six months and to regain the pre-pregnancy weight. Other practices that need to be promoted are self-monitoring of blood glucose, MNT, regular exercise, antenatal classes, contraception, pregnancy spacing, and health care between pregnancies.

### Modify cultural beliefs and practices that may contribute to GDM

Some ethnic groups believe that pregnancy induces a craving for sugary foods. E.g., Chinese women may consume red date drinks frequently to improve blood circulation and increase iron. These drinks may contain high levels of sugar which can affect blood glucose level, thereby contributing to GDM.

Increase interventions to promote health literacy and diabetes education, empowerment of women (through adult education), self-care about GDM, knowledge of the intergenerational incidence of DM. Education should emphasize the importance of a healthy lifestyle at all levels, from the young to the elderly population, and included it in the school curriculum.

### Intensify research in the following areas

- The role of iron supplementation on the risk of iron toxicity and GDM among women with genetic hemoglobin disorders, particularly in countries where the condition is prevalent.
- The role of micronutrient deficiencies in relation to GDM and whether ethnic-specific micronutrient requirements are needed.
- The role of MNT in GDM management. Local research based on ethnic diversity and food habits should be carried out for the development of individualized meal plans. Countries should develop local dietary guidelines, diet therapy, and culturally appropriate meal plans for GDM based on local research rather than simply adopting findings from non-Asian populations.
- National figures on normal weight gain in pregnancy are lacking. Appropriate gestational weight gain in Asian women need to be determined, depending on pre-pregnancy BMI. Factors that influence postpartum weight and ways to lose excess weight should be studied. In Malaysia and Vietnam, the majority of mothers retained their weight at 6-months postpartum.
- Determinants of health status in pre-pregnant and pregnant Asian women, the nutrigenomics and nutrigenetics of women likely to develop GDM.
- Effective nutrition education and communication strategies to achieve behavior change, including culturally appropriate approaches that encourage overweight women to come for consultation without increasing pressure or reducing their self-esteem.

## Conclusion

The ILSI symposium highlighted the need for urgent actions to address the growing problem of GDM in Southeast Asia. Recommendations include the use of common regional screening methods and diagnostic criteria for GDM, generation of national and regional epidemiologic data on GDM prevalence and predisposing factors, and stimulation of local research to identify best practices for prevention and management of GDM in specific countries.
